# Identifying and Estimating Frailty Phenotypes by Vocal Biomarkers: Cross-Sectional Study

**DOI:** 10.2196/58466

**Published:** 2024-11-08

**Authors:** Yu-Chun Lin, Huang-Ting Yan, Chih-Hsueh Lin, Hen-Hong Chang

**Affiliations:** 1 Graduate Institute of Integrated Medicine College of Chinese Medicine China Medical University Taichung Taiwan; 2 Department of Chinese Medicine China Medical University Hospital Taichung Taiwan; 3 Institute of Political Science Academia Sinica Taipei Taiwan; 4 School of Medicine College of Medicine China Medical University Taichung Taiwan; 5 Department of Family Medicine China Medical University Hospital Taichung Taiwan; 6 Chinese Medicine Research Centre China Medical University Taichung Taiwan

**Keywords:** frailty phenotypes, older adults, successful aging, vocal biomarkers, frailty, phenotype, vocal biomarker, cross-sectional, gerontology, geriatrics, older adult, Taiwan, energy-based, hybrid-based, sarcopenia

## Abstract

**Background:**

Researchers have developed a variety of indices to assess frailty. Recent research indicates that the human voice reflects frailty status. Frailty phenotypes are seldom discussed in the literature on the aging voice.

**Objective:**

This study aims to examine potential phenotypes of frail older adults and determine their correlation with vocal biomarkers.

**Methods:**

Participants aged ≥60 years who visited the geriatric outpatient clinic of a teaching hospital in central Taiwan between 2020 and 2021 were recruited. We identified 4 frailty phenotypes: energy-based frailty, sarcopenia-based frailty, hybrid-based frailty–energy, and hybrid-based frailty–sarcopenia. Participants were asked to pronounce a sustained vowel “/a/” for approximately 1 second. The speech signals were digitized and analyzed. Four voice parameters—the average number of zero crossings (A1), variations in local peaks and valleys (A2), variations in first and second formant frequencies (A3), and spectral energy ratio (A4)—were used for analyzing changes in voice. Logistic regression was used to elucidate the prediction model.

**Results:**

Among 277 older adults, an increase in A1 values was associated with a lower likelihood of energy-based frailty (odds ratio [OR] 0.81, 95% CI 0.68-0.96), whereas an increase in A2 values resulted in a higher likelihood of sarcopenia-based frailty (OR 1.34, 95% CI 1.18-1.52). Respondents with larger A3 and A4 values had a higher likelihood of hybrid-based frailty–sarcopenia (OR 1.03, 95% CI 1.002-1.06) and hybrid-based frailty–energy (OR 1.43, 95% CI 1.02-2.01), respectively.

**Conclusions:**

Vocal biomarkers might be potentially useful in estimating frailty phenotypes. Clinicians can use 2 crucial acoustic parameters, namely A1 and A2, to diagnose a frailty phenotype that is associated with insufficient energy or reduced muscle function. The assessment of A3 and A4 involves a complex frailty phenotype.

## Introduction

Frailty is a prevalent geriatric syndrome characterized by age-dependent declines in the functioning of multiple organ systems, resulting in elevated susceptibility to stressors and a greater likelihood of adverse health outcomes [1] and mortality [2]. In population-based studies, the global prevalence of frailty among people older than 50 years is estimated to range from 12% to 24% [3].

Frailty can be roughly categorized as physical, cognitive, and social. Researchers have developed a variety of indices to assess physical frailty, including the Frailty Index scale [4]; the Clinical Frailty Scale [5]; the Cardiovascular Health Study (CHS) index [6]; the Study of Osteoporotic Fractures index [7]; and the Fatigue, Resistance, Ambulation, Illness and Loss of weight index [8]; among others. The components of the frailty scale can be clustered. There are 2 areas of investigation that are particularly important. One area used the Frailty Index scale to identify individuals who were multifrail, cognitively and functionally frail, psychologically frail, and physiologically frail. This was accompanied by examining socioeconomic factors, immunoscenescence markers, and inflammatory biomarkers associated with distinct frailty subtypes [9,10].

Another avenue of inquiry centers on the 5 phenotypic criteria proposed by Fried et al [6] (the CHS index), which are intended to distinguish 2 [11] or 3 distinct subgroups of physical frailty [12,13]. The MacArthur Study of Successful Aging captured 2 subdimensions of the CHS phenotype, in which slower gait, weaker grip strength, and lower physical activity define the first component that can better predict cognitive impairment, disability, and mortality, while exhaustion and weight loss define the second component [11]. The I-Lan Longitudinal Aging Study and the National Institute for Longevity Sciences-Longitudinal Study of Aging distinguished among 3 subgroups: nonmobility-type, mobility-type frailty, and low physical activity; they confirmed that the mobility subtype was associated with significantly adverse outcomes [12,13]. Recent research compared the longitudinal trajectories of distinct prefrailty (PF) groups based on the CHS frailty components and reported that the PF2—defined by 1 or 2 among weakness, slowness, and low physical activity, in the absence of exhaustion and unexplained weight loss—had a higher risk of difficulty in carrying out instrumental activities of daily living and mortality than PF1—defined by exhaustion and/or unexplained weight loss, in the absence of weakness, slowness and low physical activity [14,15].

Recent research has examined several physical frailty scales with overlapping components and has distinguished these components into 2 groups: energy-based frailty (EBF) and sarcopenia-based frailty (SBF). This indicates that dietary sodium restriction is associated with a significantly increased risk of SBF, suggesting a potential pathway linking dietary sodium restriction, poor appetite, compromised nutritional status, and SBF in older adults. However, no significant association is observed between dietary sodium restriction and the likelihood of EBF [16].

Inflammation, oxidative stress and antioxidants, coagulation and platelet function, growth factors, musculoskeletal and cardiac function, amino acids and vitamins, hepatic and renal metabolism, DNA, RNA, and miRNA are potential biomarkers or alternatives of frailty [17]. A meta-analysis suggested that C-reactive protein, hemoglobin, albumin, 25-hydroxy vitamin D, and free testosterone are circulating biomarkers strongly associated with clinical frailty. However, none of them alone are sufficient for the diagnosis of frailty. That is consistent with the definition of frailty as a multidimensional syndrome caused by multiple biological alterations [18]. Digital biomarkers, such as wearable sensors recording physical activity features, were limited and may not represent all frailty phenotypes [19]. Indeed, most of these proposed biomarkers necessitate invasive, time-consuming, and relatively high-cost analytical techniques and clinical settings [20].

Voice, which is an emerging indicator of physical and mental health, has been scarcely discussed in the context of frailty. In contrast to other biomarkers, voice analysis presents a rapid, noninvasive, and cost-effective estimation tool, thereby opening up novel avenues for diagnosis, risk prediction, and remote monitoring of patients. Vocal signals feature a multisystemic physical function; they are consistent with the definition of frailty as a multidimensional syndrome and are more advanced biomarkers of frailty. Previous studies have identified acoustic features for detecting cognitive impairment [21-23], screening major depressive disorder [24-26], diagnosing diabetes mellitus [27], monitoring patients with heart failure [28,29] and pulmonary hypertension [30], and identifying patients with respiratory conditions [31]. Two recent studies have demonstrated a correlation between acoustic measures and frailty [32,33]. One study found that the most frail participants exhibited greater speech irregularity but did not have a lower voice intensity than the less frail participants [33]. Another study suggested that spectral-domain voice parameters may be potentially useful in frailty assessment, as the voice characteristics of frail older adults revealed significant variations in the first and second formant frequencies, as well as an energy increase in the low-frequency portion [32]. These results indicate that voice parameters differed according to the frailty status.

The frailty phenotype is rarely discussed in the literature on the aging voice. Nonetheless, vocal biomarkers that elucidate various physical functions and mental status may be potentially applicable in estimating diverse dimensions of frailty. Given the potential relationship and limited evidence, this study aimed to examine the potential phenotypes of frail older adults and determine their relationship with acoustic parameters.

## Methods

### Study Design and Sample

Participants older than 60 years at the geriatric outpatient clinic of a teaching hospital in central Taiwan between January and December 2020 were recruited. Participants with acute infections and inflammatory diseases (eg, laryngopharyngitis and upper respiratory tract infection), anatomic lesions of the laryngopharynx, gastroesophageal reflux disease, neurologic diseases associated with voice disorders (eg, Parkinson disease and myasthenia gravis), or a surgical history involving the neck were excluded. Eligible individuals were invited to participate in an observational study focusing on vocal biomarkers of frailty at the geriatric outpatient clinic of a teaching hospital.

### Ethical Considerations

The consent form was thoroughly reviewed and signed by all individuals who expressed their willingness to participate in the study, with the assistance of our research team members. Participants had the ability to opt out anytime during the research period. Collected data were deidentified; for example, the participants’ names and medical record numbers were replaced by a temporary ID. The study did not provide any specific compensation due to the absence of any invasive intervention. No images or biometric identifiers of individual participants are provided. The study was carried out in accordance with tenets of the Declaration of Helsinki and was approved by the Ethics Committee of China Medical University Hospital, Taiwan (CMUH108-REC3-160).

### Measurement of Frailty Phenotypes

#### Overview

Two frailty phenotypes were identified: EBF and SBF [16]. The study identified 2 additional and potential intermediate forms of the 2 frailty phenotypes: hybrid-based frailty–energy (HBF-E) and hybrid-based frailty–sarcopenia (HBF-S). The measures used to characterize the frailty phenotype are as follows.

#### EBF: the EBF Index

Weight loss was defined as unintentional weight loss of at least 4.5 kg or >5% of the body weight in the previous year. Fatigue or exhaustion was measured using the question (“In this last week, do you feel that you have less energy to do the things you want?”) and categorized as 0 (a “no” answer) or 1 (a “yes” answer). Participants were considered frail if they fulfilled 2 criteria, prefrail if they fulfilled 1 criterion, and robust if no criterion was fulfilled.

#### SBF: the SBF Index

Low resistance was assessed by measuring the ability to rise from a chair 5 consecutive times without using the arms. Low handgrip strength was assessed by measuring handgrip strength using cutoff values (for the dominant hand) modified for Asian individuals (28 kg for men and 18 kg for women) [34]. Low walking ability was evaluated on the basis of the time spent in walking a 4-m distance, with slow gait defined as a gait speed of <1.0 m/second according to the 2019 Asian Working Group for Sarcopenia [35]. Participants who could not perform the walking test, such as wheelchair users, were classified as having low mobility.

Low physical activity was assessed on the basis of the incidence and progression of basic activities of daily living disability from an emergency geriatric assessment [36], with the following question: “In last year, do you have any deterioration in activities of daily living (feeding, hygiene, dressing, transferring, walking, toileting, and bathing)?” Participants who had difficulty performing at least one of the activities were considered not physically active. Participants were considered frail if at least 3 of the 4 criteria were fulfilled, prefrail if only 1 or 2 criteria were fulfilled, and robust if none of the criteria were fulfilled.

#### HBF-E

Participants were deemed frail if they were identified as frail based on the EBF index and prefrail or frail based on the SBF index.

#### HBF-S

Participants were deemed frail if they were identified as frail based on the SBF index and prefrail or frail based on the EBF index.

### Acoustic Parameters

After relaxing for 5 minutes, participants sitting in a separate quiet room and maintaining about a 10-cm distance between their mouth and a 90° angle unidirectional stereo condenser microphone (SONY ECM-MS907) were asked to pronounce a sustained vowel “/a/” for approximately 1 second with natural speech. Then, the speech signals were recorded and digitized using a sound blaster (SB1090, Creative Labs) at a sampling rate of 44.1 kHz with an antialiasing function and analyzed using LabVIEW (National Instruments). An end point–detecting algorithm was incorporated to eliminate the leading and trailing nonspeech portions of each utterance [37]. A recording signal that was either shorter than 0.8 seconds or longer than 1.2 seconds was withdrawn. For the calibration and validation of recording equipment, the analyzing program included a standard vowel “/a/” sound, which was used to help participants pronounce a sustained vowel “/a/” correctly. Only parameters with a variability less than 15% could be further analyzed by repeating the test 3 times. Researchers also observed sound waveforms and parameters to identify inappropriate signals and asked participants to rerecord the sounds. The manufacturer conducted periodic calibrations of the device.

The study applied 4 voice parameters—the average number of zero crossings (A1), variations in local peaks and valleys (A2), variations in the first and second formant frequencies (A3), and spectral energy ratio (A4)—to analyze voice changes [37]. A1 was defined as the number of times the signal changed in value, from positive to negative, and vice versa, divided by the frame length. A2 was calculated as the average deviation of the largest (and the smallest) values for all peaks (and valleys), as a reflection of the degree of the temporal stability of vocal variations. A3 was defined as the average deviation from the mean of the first and second formant frequencies, which depend on vocal tract length and the location and narrowness of constrictions along the vocal tract. A4 was defined as the ratio of the spectral energy below 800 Hz (end frequency) to the total spectral energy.

### Statistical Analysis

Statistical analyses were performed using the Stata (StataCorp) software. Descriptive statistics were used to describe the study data, including absolute and percentage frequency distributions, mean, and SD. The chi-square test and 1-way ANOVA were respectively used to compare categorical and continuous variables, with *P*<.05 indicating statistical significance. Each acoustic variable was separately evaluated in relation to the frailty phenotype. Logistic regression was used for elucidating the prediction model. Binomial logistic regression was used in situations where the outcome of a target variable can be limited to only 2 distinct types (eg, EBF versus non-EBF). Odds ratios (ORs) and 95% CIs of the variables included in each model were calculated. The odds to predicted probabilities were also converted using the following formula: probability = odds / (1 + odds). A multinomial logistic regression model was applied to predict the probabilities of the various possible outcomes of a multicategorical dependent variable (eg, nonfrail, EBF, SBF, and both types of frailties). We reported relative risk ratios (RRRs), defined as the ratio of the probability of choosing one outcome category to that of choosing the baseline comparison group.

## Results

### Characteristics of Nonfrail and Frail Participants

**Figure 1:**
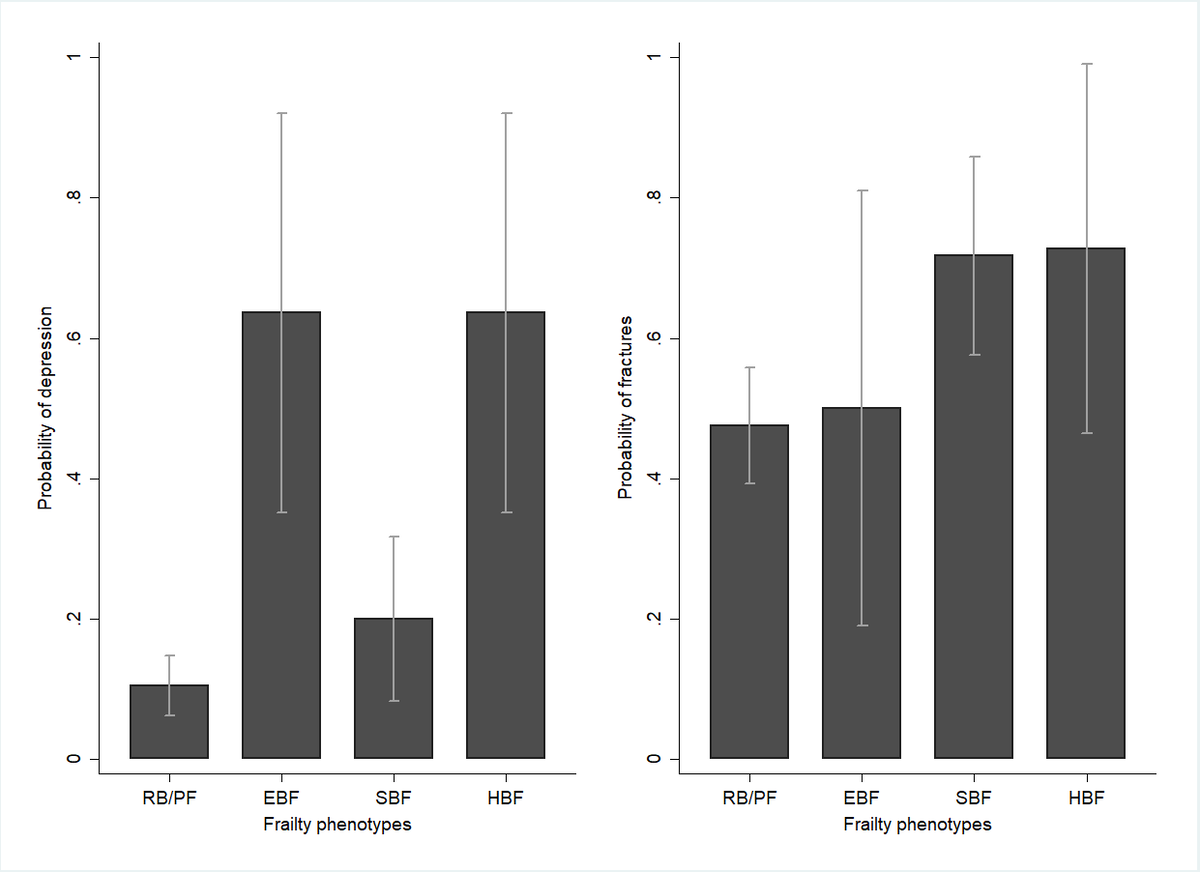
The association between frailty phenotypes and the probability of depression and fractures among older adults in 2020.

A total of 277 older adults were assessed. Frailty, as defined by the EBF index, was associated with reduced body weight and BMI, malnutrition, and depression, whereas frailty, as defined by the SBF index, was associated with older age, dementia, and fractures. The 4 frailty phenotypes were associated with polypharmacy and falls (Table S1 in Multimedia Appendix 1). When the participants were classified into 4 categories (nonfrail, EBF, SBF, and both types of frailty) and multinomial logistic regression was used, the risk of depression was higher among those with only EBF (RRR 14.92, *P*<.001), while fractures were more likely among those with only SBF (RRR 2.81, *P*=.009; Figure 1). These results suggest differences in the characteristics between nonfrail and frail older people.

### Acoustic Features and Frailty Phenotypes

**Figure 2:**
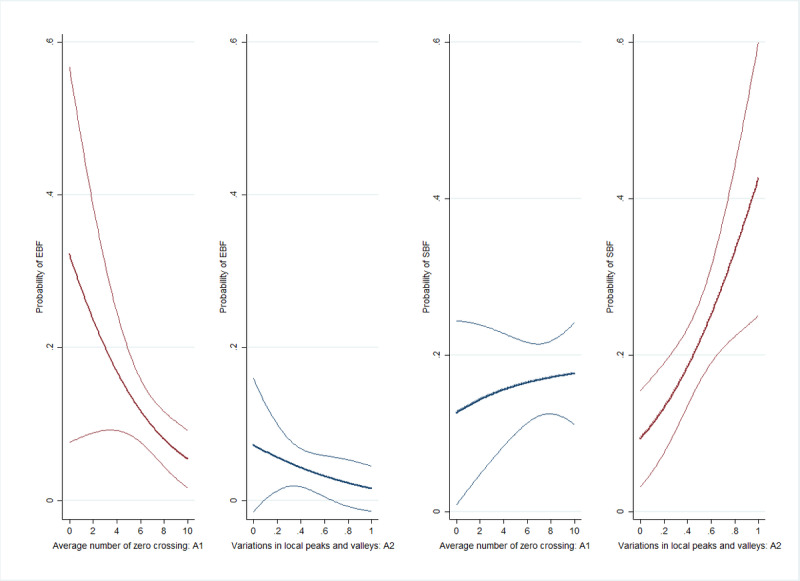
The association between acoustic features and the probability of energy-based frailty (EBF) and sarcopenia-based frailty (SBF) among older adults in 2020.

The acoustic features were related to the probability of frailty as defined by the EBF and SBF indices among older adults (Figure 2). An increase in A1 values was associated with a lower likelihood of EBF (OR 0.81, 95% CI 0.68-0.96), whereas an increase in A2 values resulted in a higher likelihood of SBF (OR 1.34, 95% CI 1.18-1.52). When the participants are classified into 4 categories (nonfrail, EBF, SBF, and both types of frailty) and multinomial logistic regression was used, no significant association was found between A1 and the likelihood of SBF (RRR 0.89, 95% CI 0.66-1.20), and between A2 and the likelihood of EBF (RRR 1.01, 95% CI 0.89-1.15).

**Figure 3:**
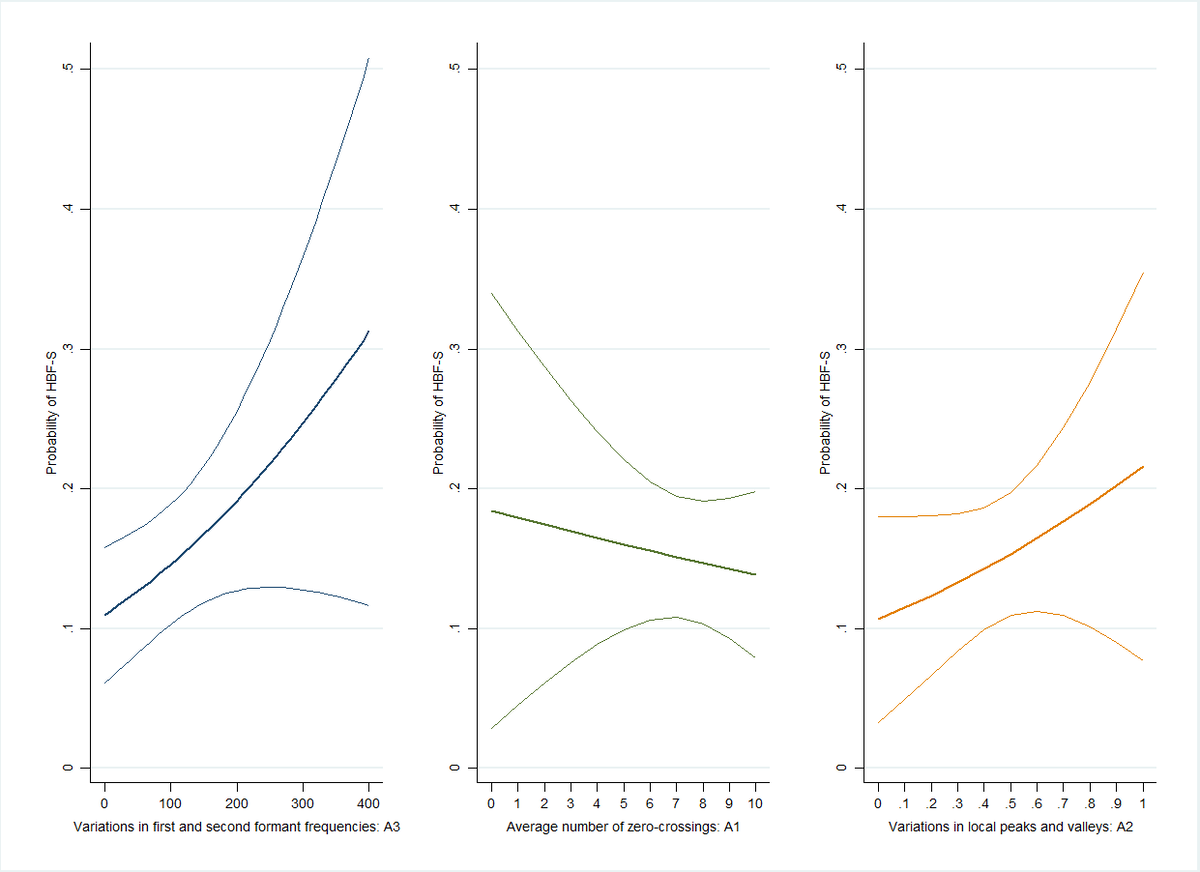
The association between acoustic features and the probability of hybrid-based frailty–sarcopenia (HBF-S) among older adults in 2020.

**Figure 4:**
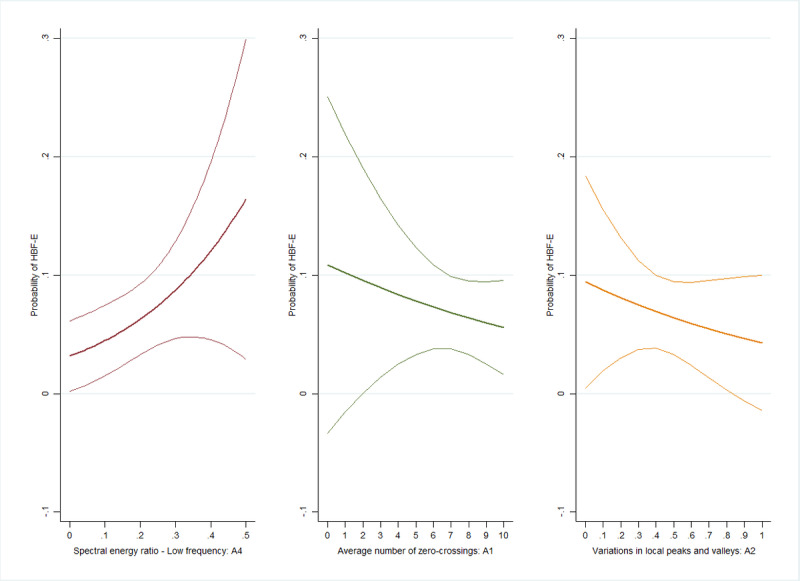
The association between acoustic features and the probability of hybrid-based frailty–energy (HBF-E) among older adults in 2020.

Respondents with higher A3 values had a higher likelihood of HBF-S (OR 1.03, 95% CI 1.002-1.06; Figure 3). When the same criteria for HBF-S were applied, we found that the association between A1 or A2 and the likelihood of HBF-S was not significant (A1: OR 0.97, 95% CI 0.84-1.11; A2: OR 1.09, 95% CI 0.94-1.26). Instead, respondents with larger A4 values had a higher likelihood of HBF-E (OR 1.43, 95% CI 1.02-2.01; Figure 4). Similarly, no significant association was found between A1 or A2 and the likelihood of HBF-E (A1: OR 0.93, 95% CI 0.76-1.13; A2: OR 0.92, 95% CI 0.73-1.15).

Finally, when the participants were categorized as above, we did not find a significant association between A3 and the likelihood of EBF (RRR 1.02, 95% CI 0.97-1.08) or SBF (RRR 1.00, 95% CI 0.96-1.03). Similar results were observed when A4 was analyzed (EBF: RRR 1.33, 95% CI 0.86-2.07; SBF: RRR 1.05, 95% CI 0.81-1.38).

## Discussion

### Principal Findings

To the best of our knowledge, this is the first study to ascertain the correlation between frailty phenotypes and acoustic parameters among older adults. Our study identified 4 distinct frailty phenotypes, with A1 being better at identifying older adults with EBF and A2 being much better at identifying older persons with SBF. A3 and A4 can serve as reliable predictors for individuals who report prefrailty or frailty in one dimension and frailty in another dimension.

A1 might reflect the airflow volume initiated by lung contraction during phonation to which the component of exhaustion and weight loss are more related [38]. Prior to speaking, individuals engage in deep breathing to expand their lung volume. Subsequently, a notable increase in esophageal pressure occurs during the expiratory phase, coinciding with the initiation of airflow for speech production [39]. Frailty and respiratory impairment are strongly linked with each other [40]. Wijnant et al [41] evaluated participants with impaired pulmonary function (revealed through spirometry) and reported that they more often developed frailty with poor reversibility. Furthermore, frailty was associated with airflow obstruction and dyspnea in patients with chronic obstructive pulmonary disease [42]. A study on the association between frailty syndrome and acoustic measures of voice quality and voice-related handicap suggested that the components of frailty most related to the Voice Handicap Index-10—which mainly measures voice disorders due to reduced vocal loudness or increased vocal effort from limited airflow [43]—were exhaustion and weight loss rather than slowness, weakness, or low physical activity [44]. In fact, exhausted older adults complain of reduced vocal loudness or increased phonatory effort [45]. These results are consistent with our findings that participants who were more prone to fatigue and body weight loss performed fewer zero crossings on average (A1).

A2 might indicate glottal flow stability during phonation, which is attributed to the control of muscle mass and strength [38]. During the initial phase of speech, the inspiratory external intercostal muscles come into play, effectively opposing the passive recoil of the thorax and lungs. However, as the respiratory cycle progresses, the expiratory internal intercostal muscles take precedence, ensuring a consistent level of air pressure within the trachea [39]. Thus, impaired aerodynamic force control causes greater expansion of the chest and lungs and necessitates more abdominal movement to increase vocal amplitude, which, in turn, results in larger variations in the local peaks and valleys (A2). Indeed, age-related dysphonia is primarily attributed to the weakness of respiratory, laryngeal, and lingual muscles [45]. Previous research suggested that karaoke training exercises may be a key to increase activity in the respiratory muscles, thus improving pulmonary function and reducing chest stiffness [46]. Additionally, exercises strengthening suprahyoid muscles have demonstrated greater compensatory effects on swallowing and voice production [47]. Poor vocal cord control via the laryngeal muscles also plays a role in aerodynamic instability. The loss of muscle mass and strength could potentially exert a significant effect on the development of presbylarynx, that is, the loss of vocal fold tone and elasticity with aging. In fact, Santos et al [48] reported a correlation between functional impairment and presbylarynx. These results are consistent with our findings that components of SBF, including grip strength, walking speed, physical activity, and chair stands, which reflect muscle mass and strength, are more related to A2.

Frequency-domain parameters (A3 and A4) are better acoustic predictors of a complex frailty phenotype, of which A3 can evaluate frailty that is closer to the sarcopenia type, while A4 can assess frailty that is closer to the energy type. According to the source-filter theory, changes in vocal tract shape, including tongue, laryngeal, and mouth adjustments, are made to produce resonances to specific frequencies [49]. Fine muscular control as well as extensive neurological involvement are required to produce acoustic characteristics of vowels and some consonants during the phonation process [50]. Acoustic parameters—variations in the first and second formant frequencies (A3)—may be determined by both muscular and glottal flow control where the former plays a major role.

A4 may also be related to the interplay of glottal airflow and muscular control of the vocal fold. A plausible major mechanism is that reduced glottal flow causes an increase in the time spent in opening the glottis, which subsequently leads to a more dominant first harmonic in the low-frequency portion of the voice source spectrum, thus increasing the energy in the low-frequency portion of the source spectrum [51]. In breathy (hypofunctional) phonation, relatively low values of the maximum flow declination rate—the major determinant of the peak amplitude and energy portion of the produced voice—from the glottal area waveform were found [52]. Furthermore, the loss of mass and strength in thyroarytenoid muscles that control the vocal cords may result in reduced vertical thickness of the vocal cord and contribute to a longer open phase of the glottis, thereby generating more predominant low-frequency energy (A4) [38]. However, further evidence is required to support these claims.

These findings indicate that older adults with EBF are likely to report depression, whereas those with SBF are likely to have fractures. This finding is in line with previous evidence. Weight loss is associated with an increase in depressive symptoms [53]. Furthermore, older adults with involuntary weight loss are significantly more likely to experience fatigue, as physical fatigue might be partly due to a lack of energy or nutrients [54]. Fatigue is associated with a greater likelihood of deterioration in subsequent self-rated health, functional status, loneliness, depression, and physical activity level [55]. EBF is characterized by involuntary weight loss and fatigue, which suggests a potentially strong association with depressive symptoms. Several studies suggest that low handgrip strength [56], low walking ability [57], and low physical activity [58] are associated with the risk of fractures. This suggests that SBF is potentially strongly associated with fractures, as it exhibits low muscle strength, walking ability, and physical activity.

### Limitations

This study has several limitations. First, some acoustic characteristics are not captured within a specific time frame, despite the fact that the sustained vowel “/a/” possesses the advantage of pronunciation without training [37], resulting in a greater overall intensity of voice and elevating the level of reproducible assessment [59], thus rendering it relatively stable for analysis. Future studies can allow participants to pronounce distinct vowels or modify the program to record their voices for different time frames to determine whether this would affect the results. Second, dynamic adjustments to voice frequency and amplitude are required, given that steady vowel utterances bear limited resemblance to natural language production [60]. Nonetheless, biomarkers A1 to A4 proved to be more advantageous in analyzing the changes in the voice than commonly used voice parameters, such as pitch frequency or pitch variations, volume of the voice, and speed of speech, given that the results were unaffected by psychological factors [37]. Third, there is a dearth of data pertaining to the mechanisms that link the frailty phenotypes to the alteration in glottal airflow and anatomical changes that result in alterations in the acoustic properties of voice. More physiological data may help determine causality. Fourth, patient recruitment from a single center may limit the external generalizability of our findings. Multicenter studies should be the focus of future research, allowing for faster recruitment, diverse population coverage, and enhanced generalizability. Fifth, confounding factors, such as recording time (in the morning or afternoon), race, and other physical and mental health conditions, may affect acoustic characteristics, which may bias the outcomes. However, no existing data are available to confirm this. Future research should examine confounding effects in the event that additional data become available. Finally, this study identified distinct frailty phenotypes mainly based on the physical dimension of frailty, thereby failing to use psychological and social components of frailty for developing a holistic typology and corresponding definitions of frailty. Future research that considers such issues would enrich our understanding of the link between diverse frailty phenotypes and voice-related measures.

### Implications in Clinical Practice and Diagnostics

Two implications for clinical practice deserve consideration. First, efforts to link acoustic measures to the diagnosis of frailty phenotypes in older adults could commence with 2 crucial acoustic parameters, namely A1 and A2, which roughly indicate insufficient energy or reduced muscle function. Second, the assessment of A3 and A4 involves a complex frailty phenotype that is associated with involuntary loss of body weight, fatigue, and loss of muscle mass and function.

Remote monitoring of physical frailty is crucial for personalized care to decelerate overall deterioration and/or promote the healthy recovery of older adults following exposure to acute or chronic stressors. For example, the monitoring of frailty status in patients with chronic conditions such as cerebrovascular accident or diabetes in home health care and long-term care institutions can improve the prognosis and treatment of chronic diseases. The application of this program in annual health checkups for older adults, geriatric integrated outpatient clinics, and cardiac and pulmonary rehabilitation workflow in clinical practice may be explored in future research. Furthermore, future research may integrate this program into smartphone apps, vocal chatbots, voice assistants, or other smart devices to record a simple vowel sound for remote monitoring in clinical practice and telemedicine [61].

### Conclusions

Given that the frailty phenotype is seldom discussed in the literature on the aging voice, this study, to the best of our knowledge, is the first to determine the relationship between frailty phenotypes and acoustic parameters among older adults. As a noninvasive, instantaneous, objective, and cost-effective estimation tool, the 4 vocal biomarkers have the potential to assess distinct frailty phenotypes.

## Appendix

Multimedia Appendix 1 Characteristics of robust/prefrail and frail participants based on the energy-based frailty index, sarcopenia-based frailty index, hybrid-based frailty–energy index, and hybrid-based frailty–sarcopenia index.

## Abbreviations

A1: average number of zero crossings
A2: variations in local peaks and valleys
A3: variations in first and second formant frequencies
A4: spectral energy ratio
CHS: Cardiovascular Health Study
EBF: energy-based frailty
HBF-E: hybrid-based frailty–energy
HBF-S: hybrid-based frailty–sarcopenia
OR: odds ratio
PF: prefrailty
RRR: relative risk ratio
SBF: sarcopenia-based frailty

## Data Availability

The datasets used and/or analyzed in this study are available from the corresponding author on reasonable request.
