# Supplementary material for: Identifying and Estimating Frailty Phenotypes by Vocal Biomarkers: Cross-Sectional Study
Source: J Med Internet Res. 2024 Nov 8;26:e58466. doi: 10.2196/58466 (PMC11584546; doi:10.2196/58466)
Supplement: Multimedia Appendix 1 [file jmir_v26i1e58466_app1.docx]

Table S1. Characteristics of robust/prefrail and frail participants based on the energy-based frailty (EBF) index, the sacropenia-based frailty (SBF) index, the hybrid-based frailty (energy) (HBF-E) index, and the hybrid-based frailty (sacropenia) (HBF-S) index.

|  | EBF | | | | | HBF-E | | | | | HBF-S | | | | | SBF | | | | |
| --- | --- | --- | --- | --- | --- | --- | --- | --- | --- | --- | --- | --- | --- | --- | --- | --- | --- | --- | --- | --- |
|  | Robust/  prefrail | | Frail | |  | Robust/  prefrail | | Frail | | | Robust/  prefrail | | Frail | | | Robust/  prefrail | | Frail | | |
| N= 277 | N | (%) | N | (%) | P value† | N | (%) | N | (%) | P value† | N | (%) | N | (%) | P value† | N | (%) | N | (%) | P value† |
| Women | 160 | (64.8) | 14 | (50.0) | .12 | 159 | (63.9) | 7 | (38.9) | .04 | 139 | (61.2) | 27 | (67.5) | .45 | 102 | (61.8) | 64 | (62.8) | .88 |
| Age (years) ‡ | 74.2 | ±6.8 | 74.9 | ±9.1 | .62 | 74.2 | ±6.8 | 79.3 | ±7.0 | .002 | 73.7 | ±6.6 | 79.5 | ±6.6 | <.001 | 72.5 | ±6.4 | 77.7 | ±6.6 | <.001 |
| Economic status | 17 | (7.0) | 1 | (3.6) | .49 | 37 | (14.8) | 4 | (22.2) | .82 | 11 | (5.0) | 7 | (17.5) | .004 | 11 | (6.8) | 7 | (7.1) | .93 |
| Body weight (kg) ‡ | 59.1 | ±10.4 | 48.3 | ±8.1 | <.001 | 59.1 | ±10.4 | 46.6 | ±6.4 | <.001 | 59.3 | ±10.0 | 52.4 | ±12.2 | <.001 | 59.2 | ±10.2 | 56.8 | ±11.2 | .07 |
| BMI (kg/m^2^) ‡ | 24.3 | ±4.1 | 20.1 | ±2.8 | <.001 | 24.3 | ±4.1 | 19.7 | ±2.4 | <.001 | 24.1 | ±4.0 | 23.0 | ±4.7 | .10 | 23.9 | ±4.0 | 24.0 | ±4.3 | .89 |
| Polypharmacy (>8 kinds) | 16 | (6.5) | 11 | (39.3) | <.001 | 19 | (7.6) | 8 | (44.4) | <.001 | 13 | (5.7) | 14 | (35.0) | <.001 | 3 | (1.8) | 24 | (23.5) | <.001 |
| Depression | 30 | (12.2) | 20 | (71.4) | <.001 | 34 | (13.7) | 10 | (55.6) | <.001 | 23 | (10.1) | 21 | (52.5) | <.001 | 20 | (12.1) | 24 | (23.5) | .02 |
| Dementia | 36 | (14.6) | 5 | (17.9) | .65 | 35 | (14.1) | 5 | (27.8) | .12 | 28 | (12.3) | 12 | (30.0) | .004 | 11 | (6.7) | 29 | (28.4) | <.001 |
| Malnutrition | 86 | (35.0) | 20 | (71.4) | <.001 | 90 | (36.1) | 10 | (55.6) | .10 | 78 | (34.4) | 22 | (55.0) | .01 | 52 | (31.5) | 48 | (47.1) | .01 |
| Fall | 52 | (21.1) | 18 | (64.3) | <.001 | 54 | (21.7) | 15 | (83.3) | <.001 | 47 | (20.7) | 22 | (55.0) | <.001 | 25 | (15.2) | 44 | (43.1) | <.001 |
| Fractures | 96 | (52.5) | 13 | (48.2) | .68 | 95 | (51.6) | 13 | (76.5) | .049 | 83 | (50.3) | 25 | (69.4) | .04 | 49 | (44.1) | 59 | (65.6) | .002 |

† Chi square/ Fisher's test or ANOVA test. ‡ Mean ± standard deviation.

These characteristics were assessed using the items from the Emergency Geriatric Assessment (see Ke YT, Peng AC, Shu YM, et al. Emergency geriatric assessment: a novel comprehensive screen tool for geriatric patients in the emergency department. Am J Emerg Med. 2018;36:143–146. doi:10.1016/j.ajem.2017.07.008.)
EBF: energy-based frailty; SBF: sacropenia-based frailty; HBF-E: hybrid-based frailty (energy); HBF-S: hybrid-based frailty (sacropenia); BMI: body mass index.
